# Structural insights into the binding of zoledronic acid with RANKL *via* computational simulations

**DOI:** 10.3389/fmolb.2022.992473

**Published:** 2022-09-19

**Authors:** Ruijie Wang, Wenjie Zhang, Hailong Ma, Duohong Zou, Zhiyuan Zhang, Shaoyi Wang

**Affiliations:** ^1^ Department of Oral Surgery, Shanghai Ninth People’s Hospital, Shanghai Jiao Tong University School of Medicine, Shanghai, China; ^2^ Shanghai Key Laboratory of Stomatology, Research Unit of Oral and Maxillofacial Regenerative Medicine, National Center for Stomatology, National Clinical Research Center for Oral Diseases, College of Stomatology, Chinese Academy of Medical Sciences, Shanghai Jiao Tong University, Shanghai, China; ^3^ Department of Prosthodontics, Shanghai Ninth People’s Hospital, Shanghai Jiao Tong University School of Medicine, Shanghai, China; ^4^ Department of Oral Maxillofacial-Head and Neck Oncology, Shanghai Ninth People’s Hospital, Shanghai Jiao Tong University School of Medicine, Shanghai, China

**Keywords:** molecular simulation, BRONJ (bisphosphonate-related osteonecrosis of the jaw), molecular docking, molecular dynamic (MD) analysis, surface plamon resonance

## Abstract

Zoledronic acid (ZOL) inhibits receptor activator of nuclear factor-κB ligand (RANKL) and reduces bone turnover. This plays an important role in the development of bisphosphonate-related osteonecrosis of the jaw (BRONJ). Previous reports have shown that ZOL binds to the enzyme farnesyl pyrophosphate synthase (FPPS) to block its activity. However, the mechanism of action of ZOL and its interaction with RANKL is still unclear. In this study, we confirmed that ZOL significantly suppressed the bone remodeling in ZOL-treated rats, investigated whether ZOL could bind to RANKL and examined the interactions between these molecules at the atomic level. Surface plasmon resonance (SPR) assay was performed to validate that ZOL could directly bind to RANKL in a dose dependent manner, and the equilibrium constant was calculated (K_D_ = 2.28 × 10^−4^ M). Then, we used molecular docking simulation to predict the binding site and analyze the binding characteristics of ZOL and RANKL. Through molecular dynamics simulation, we confirmed the stable binding between ZOL and RANKL and observed their dynamic interactions over time. Binding free energy calculations and its decomposition were conducted to obtain the binding free energy −70.67 ± 2.62 kJ/mol for the RANKL–ZOL complex. We identified the key residues of RANKL in the binding region, and these included Tyr217(A), Val277(A), Gly278(A), Val277(B), Gly278(B), and Tyr215(C). Taken together, our results demonstrated the direct interaction between ZOL and RANKL, indicating that the pharmacological action of ZOL might be closely related to RANKL. The design of novel small molecules targeting RANKL might reduce the occurrence of BRONJ.

## Introduction

Zoledronic acid (ZOL), a third-generation, nitrogen-containing bisphosphonate, has been identified to significantly increase bone mineral density. One of the main side effects in patients treated with ZOL is bisphosphonate-related osteonecrosis of the jaw (BRONJ) (Aguirre et al., 2021; Zhou et al., 2021). This may be attributed to the decrease of osteoclast differentiation, survival, and bone turnover ability caused by ZOL (Giusti and Bianchi, 2015; Nagaoka et al., 2015). ZOL inhibits the enzyme farnesyl pyrophosphate synthase (FPPS), and researchers have analyzed the forces driving the formation of complexes, as well as investigated the potency of a wide range of bisphosphonates to FPPS (Dunford et al., 2001; Yin et al., 2006; Ohno et al., 2011). In the meantime, several studies have demonstrated that the pharmacological effect of ZOL is closely related to the inhibition of receptor activator of nuclear factor-κB ligand (RANKL) (Liu et al., 2016; Huang et al., 2019). It has been found that RANKL regulates bone remodeling, and is essential for the development and activation of osteoclasts (Jones et al., 2002). Jones et al. (2002) have observed that *Rankl* ^*−*/*−*^mice display severe osteopetrosis and stunted growth, and *Rankl* ^*−*/*−*^osteoblasts cannot support osteoclastogenesis. In animal studies, the administration of RANKL, an essential mediator of osteoclastogenesis, can induce high bone turnover (Li et al., 2000). Conversely, inhibiting RANKL may lead to the decrease of bone turnover (Lloyd et al., 2008). The suppression of RANKL plays an important role in the occurrence of BRONJ (Zhang et al., 2020). The association of BRONJ with potent antiresorptive drugs and the increased risk with higher doses of ZOL is consistent with this contention (Stopeck et al., 2010; Yang et al., 2019).

RANKL-induced osteoclastogenesis is initiated when RANKL binds to its cognate receptor RANK, which is expressed by pre-osteoclasts. The binding of RANKL to RANK triggers a cascade of signaling events, resulting in activation and translocation of nuclear factor-κB (NF-κB) to the nucleus, and this culminates in the expression of differentiation-inducing osteoclast-specific genes (Leibbrandt and Penninger, 2008). Previous reports have shown that ZOL inhibits the expression of RANKL on osteoblasts to restrain osteoclastogenesis (Liu et al., 2016; Huang et al., 2019). In addition, ZOL may directly decrease the physiological effects of RANKL protein in terms of inducing osteoclast differentiation, bone resorptive activity, and downregulating the expression of calcitonin receptor, tartrate-resistant acid phosphatase, and dendritic cell-specific transmembrane protein (Huang et al., 2019).

RANKL is a tumor necrosis factor superfamily molecule with 316 amino acids, and it is recognized as a type-II transmembrane protein because it has carboxy terminus outside the cell (Munasinghe et al., 2017). Three RANKL subunits assemble to form the trimeric molecule to function in an organism, it also exists in a soluble ectodomain form from proteolytic cleavage of the transmembrane form (Lam et al., 2001; Luan et al., 2012). All factors that inhibit bone resorption *via* osteoclasts act through RANKL as a key regulator (Khan et al., 2015). The anti-resorptive potency of ZOL has been known to be influenced by the chemical structure of the side chain attached to the central carbon of the phosphorus-carbon-phosphorus (P–C–P) backbone (Ohno et al., 2011). ZOL not only directly affects the resorption of osteoclasts in mature bone but also influences the osteoclast recruitment and differentiation by cleavage of transmembrane RANKL (Pan et al., 2004).

Recently, researchers have reported that ZOL acts on RANKL and inhibits osteoclast formation and bone resorption. Considering the binding interaction of complexes between ZOL and FPPS, thus we postulate that there is a certain interaction between ZOL and RANKL that results in changes in the biological function of RANKL (Soundia et al., 2021). Computational simulations may provide new insights into the mechanisms of action of ZOL and structure-activity relationship at the atomic level. After predicting and evaluating the stable binding conformation of ZOL and the target protein, the relevant key amino-acid residues can be identified, and this may be a critical step toward understanding complex biological processes.

In the present study, we first validated that ZOL significantly suppressed the bone remodeling in rat, then we applied surface plasmon resonance (SPR) assay, molecular docking, molecular dynamics (MD) simulation, and binding free energy calculations and its decomposition to validate the direct binding of ZOL to RANKL; we constructed the 3D structure of the RANKL–ZOL binding complex and examined its interaction. Key residues located in the binding regions of ZOL and RANKL were deciphered.

## Materials and methods

### Animal study

Twelve female Sprague-Dawley (SD) rats aged 8 weeks were primarily purchased from the Animal Care and Experiment Committee of Ninth Peoples Hospital affiliated to Shanghai Jiao Tong University, School of Medicine. Animals were housed in a specified-pathogens free facility and all procedures were performed in accordance with Institutional Animal Care and Use Committee approval (SH9H-2020-T37-3). Rats were randomly divided into two groups: ZOL-treated group (group A, *n* = 6) and control group (group B, *n* = 6). The ZOL-treated group were given ZOL (Sigma-Aldrich, United States, 66 μg/kg) by intraperitoneal injections three times a week for 6 weeks. The control group were given saline solution as blank control.

### Sequential fluorescent labeling

Sequential fluorescent labeling was carried out to label the mineralized tissue and assess the time course of bone remodeling. At 6, 8, and 10 weeks, the animals were intraperitoneally administered with 25 mg hydrochloride tetracycline (TE, Sigma, United States) (yellow), 20 mg calcein (CA, Sigma, United States) (green), and 30 mg alizarin red s (AL, Sigma, United States) (red) respectively. All animals were euthanized at the end of the experiment. The samples were fixed in 10% buffered formalin, dehydrated in ascending concentrations of alcohols, and embedded in polymethymetacrylate (PMMA). The specimens were cut in 150 μm thick sections using a microtome (Leica, Hamburg, Germany), and ground to a final thickness of 70 μm. Fluorescent labeling was observed under confocal laser scanning microscope (CLSM; Leica TCS Sp2 AOBS Germany). The calculated parameters were mineralizing surface (MS/BS, %), mineral apposition rate (MAR, μm/day), and bone formation rate (BFR/BS, μm3/μm2/day), as we previously reported (Wang et al., 2010).

### Surface plasmon resonance assay

The SPR experiment was performed with the Biacore T200 apparatus (Cytiva, United States) at 25°C. Recombinant human RANKL trimer protein was purchased from ACROBiosystems (United States). Pipelines and CM5 sensor chips (Cytiva, United States) were pretreated with running buffer (1 × PBS, 0.05% Tween-20, pH 7.4). We diluted the human RANKL protein to 30 μg/ml in immobilization buffer (10 mM sodium acetate, pH 4.5) and used an Amine Coupling Kit (Cytiva, United States) to activate the sensor chips to immobilize the RANKL protein. Incremental concentrations of ZOL (15.625–250 μM) were dissolved in the running buffer and injected into Fc1 and Fc2 channels at a flow rate of 30 μl/min for 60 s association and 90 s dissociation phases. We used the Biacore T200 evaluation software to analyze the equilibrium constant (K_D_) and fit the SPR assay curves to the steady-state model.

### Modelling of human receptor activator of nuclear factor-κB ligand trimer

**FIGURE 1:**
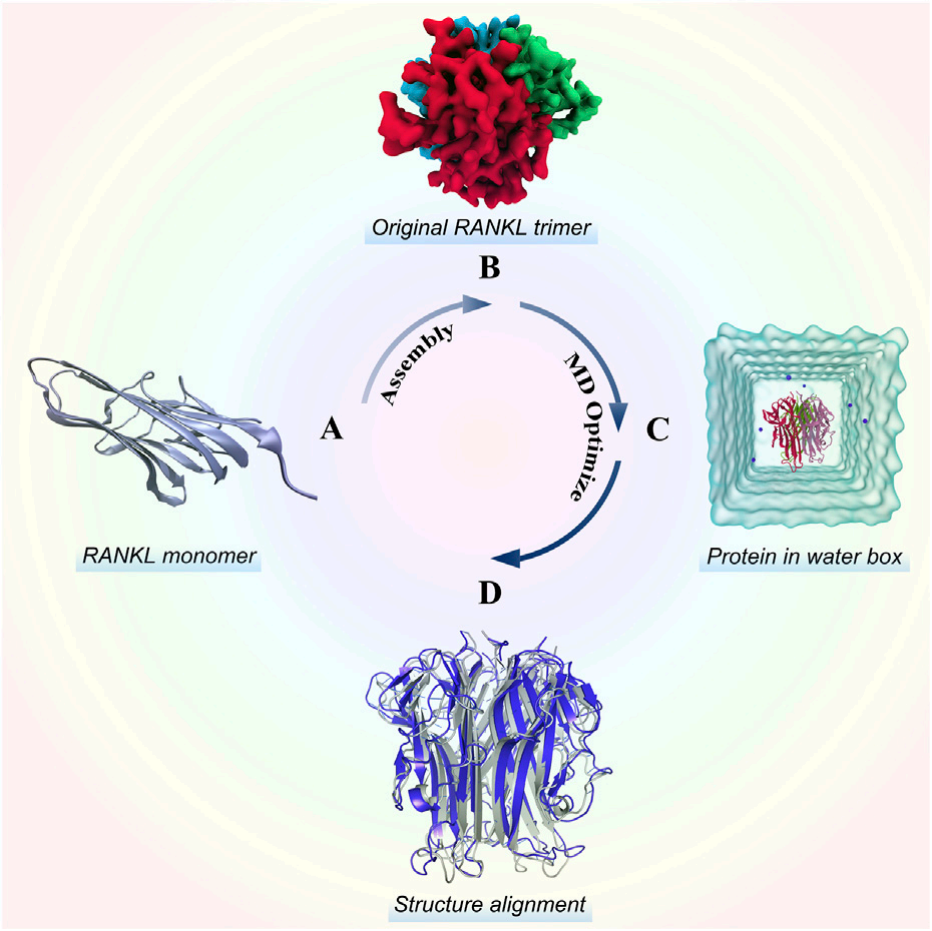
Stereo structure modelling of the RANKL trimer. **(A)** Structure of the RANKL monomer, which has a typical jellyroll-like β-sheet structure. **(B)** Original RANKL trimer structure modelled by rotating symmetric polymeric docking in M-ZDOCK online server. **(C)** Optimized RANKL trimer structure obtained by MD simulation. **(D)** Alignment of optimized structure and original structure, in which the structure colored in blue represents the optimized model and the structure colored in grey represents the original model.

As shown in Figure 1, we modeled the RANKL trimer structure using the following steps: RANKL monomer structure acquisition, trimer symmetrical assembly, and MD optimization. Then, we evaluated the quality of the structure to verify its structural reliability. For details of the modelling and quality–assessment methods, please see the Supplementary Material.

### Molecular docking

In this study, the SDF format structure file of ZOL was obtained from the PubChem database (CID_68740), and converted into the MOL2 format structure file using OpenBabel toolkit, the 2D structure was illustrated in Supplementary Figure S4. We employed ChemBio3D Ultra v12 to perform the energy minimization and stereo structure optimization of ZOL with MM2 force field. Prior to molecular docking, ZOL was prepared by adding the polar hydrogen atoms and designating Gasteiger charges, according to physiological pH 7.4. Meanwhile, we added the polar hydrogen atoms and assigned Kollman charges on RANKL as well (Wang et al., 2021; Kumar et al., 2022; Ogunyemi et al., 2022). Then we input the processed coordinate files into AutoDockTools v1.5.6 and converted this into PDBQT format files, which were needed in the follow-up docking simulation. Although the resolved structure reveals the binding interface between RANKL and OPG, it has been reported that the trimeric channels of RANKL and other proteins of the TNF superfamily may also be the potential molecular binding sites (Trang et al., 2022). The internal channel interface of RANKL trimer is an important hydrophobic region, which is also regarded as the common structural feature of TNF superfamily member proteins. The hydrophobic kernel is the crucial driving force for the homologous trimerization of RANKL (Lam et al., 2001). In addition, the drug target site of RANKL is not yet confirmed, thus we set the docking conformation searching mode to global docking covering the entire RANKL trimer to identify all possible ZOL binding sites. The grid box center coordinates were set to (40.87, 40.94, 46.30), the grid size was 54 × 64 × 56, and the grid spacing was 1 Å. AutoDock Vina v1.1.2 was used for semi-flexible molecular docking, in which the macromolecule was set as the rigid object (which cannot change its spatial shape in the docking process) and the ligand was treated as being flexible. By default, AutoDock Vina uses the iterated local search global optimization algorithm and the Broyden–Fletcher–Goldfarb–Shanno method to search for binding conformations (Trott and Olson, 2010). The binding affinities were evaluated by the semi-empirical scoring function, and a total of 20 potential binding poses were predicated. The molecular visualization packages VMD v1.9.4A53 and LigPlot^+^ v2.2.4 were used to examine the docking results.

### Molecular dynamics simulation

We selected the optimal binding conformation of ZOL and RANKL for the MD simulations which were conducted using GROMACS v2019.3. In this study, we simulated the dynamic interaction of the RANKL-ZOL complex in the system with physiological pH. The ligand ZOL was parameterized from the online database Automated Topology Builder (ATB) (http://atb.uq.edu.au/) (Stroet et al., 2018). The ATB sever can provide classical molecular force fields parameters for novel compounds and has been validated and widely employed in recent studies, including molecular simulations, computational drug design, and X-ray refinement (Subramanian et al., 2019; Yan et al., 2021). We calculated the atomic charges of ZOL at a physiological pH of 7.4. At pH = 7.4, the net charge of predominant ionization ZOL in the system was −2, where the imidazole ring (R_2_ functional group) was singly protonated and the phosphonate groups were singly deprotonated (Nancollas et al., 2006; Nieto et al., 2010; Stachnik et al., 2014) (Supplementary Figure S4). ZOL was initially optimized at the AM1 level of theory. Next, the re-optimization of the ZOL was performed at the B3LYP/6-31G* level of theory with the polarizable continuum model for water. Then, we estimated the charges of ZOL for united-atom models by fitting the electrostatic potential using the Kollman-Singh scheme. (Stroet et al., 2018). In addition, the bonded and van der Waals parameters of ZOL were taken from the GROMOS 54a7 parameter set. The gromos54A7_ATB force field, which is a new GROMOS-compatible force field and contains non-standard atom types, was selected to build the RANKL topology and position restraint files using the “pdb2gmx” module (Schmid et al., 2011). We used a cubic box as the unit cell type, and its dimensions were 9.39 nm × 9.39 nm × 9.39 nm. The protein was set centered in the simulation box and the minimum distance from the protein to the box edge was 1.2 nm. Simple point charge (SPC) model water molecules were added to the system box (Schmid et al., 2011). Since there is no net charge in the biosystem, we used the “genion” module to replace water molecules with eight Na^+^ ions to counteract the charges in the initial system to ensure its electrical neutrality. The complete system contains the RANKL–ZOL complex, 24,468 water molecules, and eight Na^+^ ions with a total of 78,330 atoms.

Before the production run, energy minimization is needed to completely relax the structure and eliminate possible steric clashes. A total of 30,000 steps energy minimization were conducted on the whole system using the steepest-descent integration algorithm. The convergence value was set to 100 kJ/(mol·nm). When the global energy minimum was reached, the position of heavy atoms was restricted, and 100-ps canonical ensemble equilibration at 300 K was performed with a constant number of particles, volume, and temperature (NVT) to deal with the system using the leap-frog integrator. Then, we performed a 100-ps isothermal-isobaric ensemble with a constant number of molecules, pressure, and temperature (NPT) for phase equilibration. At this stage, the Berendsen barostat was used to control the system pressure at 1 bar, and the time constant for pressure coupling was set to 2 ps to complete the system equilibration. In the production stage, a 200-ns non-restricted MD simulation was carried out for the system; the time step was set as 2 fs, and there was a total of 100,000,000 steps. The linear constraint solver algorithm was used to constraint the bonds to hydrogen atoms. The particle mesh ewald method was used to handle the long-range electrostatic interactions with a real-space cutoff of 1.2 nm, and the cutoff of van der Waals interactions was set as 1.4 nm (Schmid et al., 2011; Xiao et al., 2015; Nagar et al., 2021). To improve the trajectory sampling accuracy and the reliability of the research data, we carried out three independent simulations with random initial velocities for the production phase simulations. In this study, we generated the velocity according to a Maxwell distribution at the setting temperature of 300 K and used a pseudo random seed to randomize the initialized velocity.

### Calculation and decomposition of binding free energy

In this study, we extracted 150–200 ns segments of trajectory and saved it every 50 ps, total 1,000 snapshots were compacted. Then we applied the molecular mechanics/Poisson–Bolzmann surface area (MM-PBSA) method to calculate the RANKL–ZOL binding free energy ( $∆G_{binding}$ ) from three repeated MD trajectories. We used the default calculation parameters of the script, where temperature was set to 300 K and the dielectric constants of the solute and solvent were set to 2 and 80 respectively by default. The binding free energy was calculated according to the equations:

$$\begin{array}{c} ∆G_{binding}=∆G_{complex}-\left(∆G_{RANKL}+∆G_{ZOL}\right), \end{array}$$ (1)

$$\begin{array}{c} G_{binding}=∆E_{MM}+∆G_{solv}-T∆S, \end{array}$$ (2)

and the free energy of each term was estimated using:

$$\begin{array}{c} ∆E_{MM}=∆E_{int}+∆E_{vdw}+∆E_{ele}, \end{array}$$ (3)

$$\begin{array}{c} ∆G_{solv}=∆G_{PB}+∆G_{SA}, \end{array}$$ (4)

where $∆G_{complex}$ represents the total Gibbs free energy of the RANKL-ZOL complex, $∆G_{RANKL}$ and $∆G_{ZOL}$ represent their respective Gibbs free energies in solution, $∆E_{int}$ represents the internal bond energy of the molecules, $∆E_{vdw}$ represents van der Waals interactions, and $∆E_{ele}$ represents electrostatic interaction; these components make up the molecular mechanics energy of the molecule ( $∆E_{MM}$ ). The term $∆G_{solv}$ represents the solvation free energy, which contains the contributions of the polar ( $∆G_{PB}$ ) and non-polar ( $∆G_{SA}$ ) parts of the solvation free energy. In addition, it is believed that the calculation of the entropy contribution ( T∆S ) tends to have a large margin of error that introduces significant uncertainty into the result and also requires a high time cost, so the entropic contribution is generally neglected in the calculation of the binding free energy (Rastelli et al., 2010; El Bakri et al., 2021; Febres-Molina et al., 2021). We then used the script tools to perform per-residue binding-free energy decomposition to further explore the binding free-energy contribution of each residue to the interaction between ZOL and RANKL.

### Hardware information

The hardware platform used in the computational simulations of this study was an extended-ATX tower server based on dual Intel Xeon E5-2696V4 processors, 128 GB of RAM, and the Ubuntu v20.04 LTS 64-bit operating system.

### Statistical analysis

All the quantitative measurements were expressed as mean ± standard deviation (Mean ± SD). Kruskal–Wallis non-parametric procedure followed by Mann–Whitney U test was adopted for mineralizing surface, mineral apposition rate, bone formation rate analysis, *p <* 0.05 was considered to be significant. Statistical analysis was performed using the SAS 6.12 statistical software package (Cary, NC, United States).

## Results

### Analysis of sequential fluorescent labeling in rat

**FIGURE 2:**
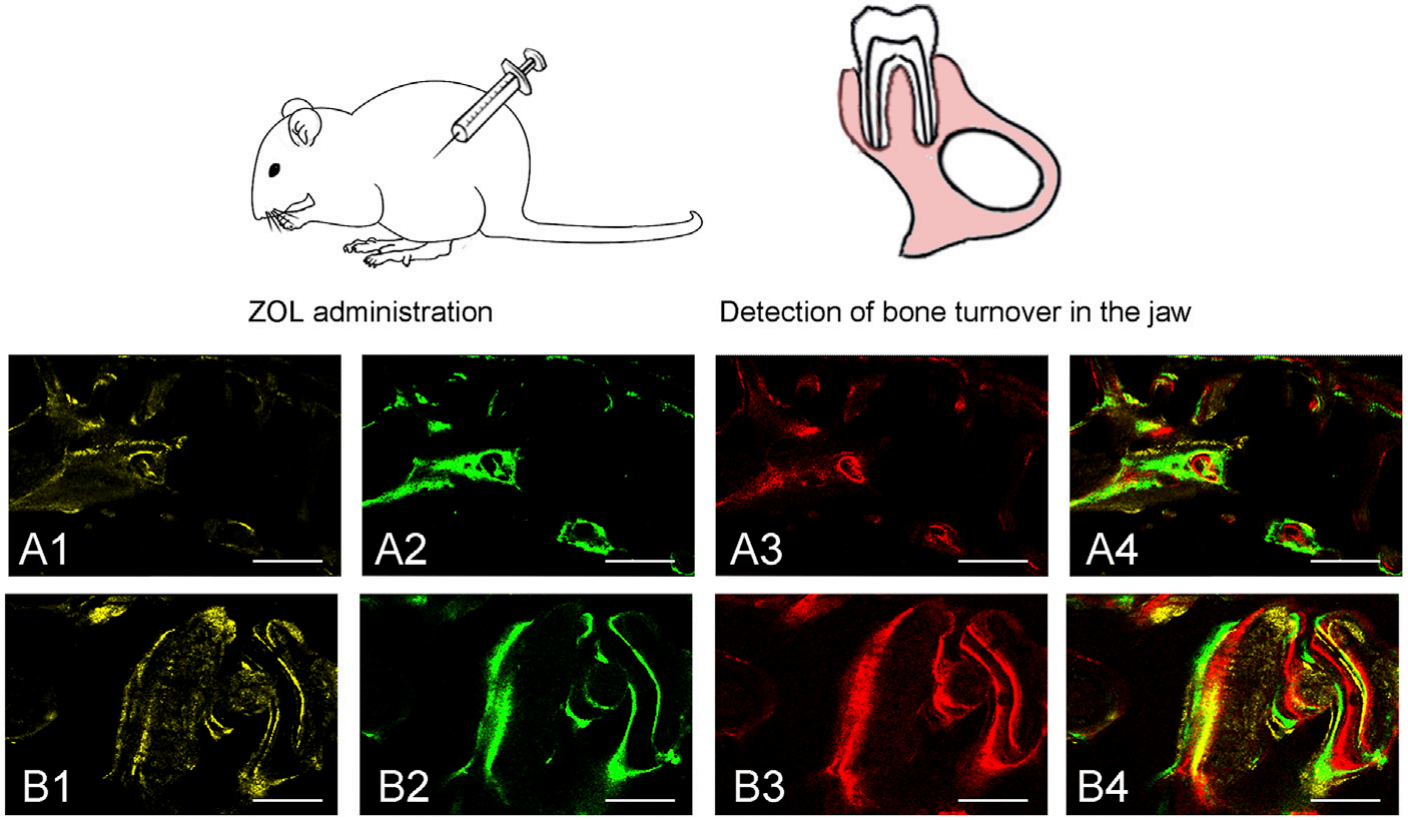
Sequential fluorescent labeling was used by TE, CA and AL at different periods. The image of (A1, A2, A3) and (B1, B2, B3) were labeled at 6, 8, and 10 weeks after ZOL/saline solution administration. A4, and B4 represented merged images of the three fluorochromes for the same group (50×). A and B represented two groups (50×).

To evaluate bone turnover in the jaw, we analyzed bone dynamic parameters. As the calculated kinetic parameters indicated that MS/BS, MAR and BFR/BS at 6–8 and 8–10 weeks were significantly lower in ZOL-treated group as compared to the control group. The data indicated that ZOL significantly suppressed the bone remodeling in group A (Figure 2; Table 1).

**TABLE 1:** The calculated parameters in bone histomorphometry.

	MS/BS (%)		MAR (μm/day)		BFR/BS (μm^3^/μm^2^/day)	
	6–8 weeks	8–10 weeks	6–8 weeks	8–10 weeks	6–8 weeks	8–10 weeks
Group A	33.44 ± 6.41	46.16 ± 5.31	0.32 ± 0.09	0.29 ± 0.05	0.42 ± 0.13	0.24 ± 0.08
Group B	50.14 ± 9.32*	62.81 ± 5.42*	0.93 ± 0.24*	0.82 ± 0.17*	0.73 ± 0.16*	0.69 ± 0.14*

Parameters are mean ± standard deviation (Mean ± SD). *Significant difference in parameters between group A and group B (*p* < 0.05).

### Direct binding between zoledronic acid and receptor activator of nuclear factor-κB ligand in surface plasmon resonance assay

**FIGURE 3:**
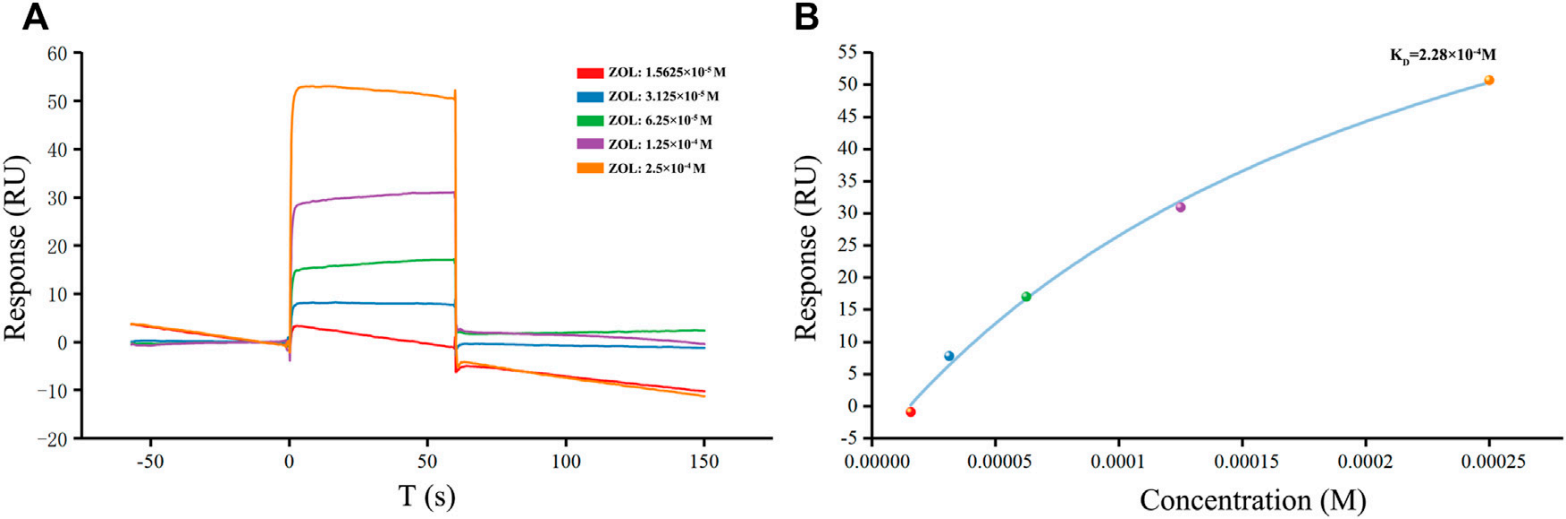
SPR detection of human RANKL and ZOL. **(A)** Plot of CM5 sensor chip containing immobilized RANKL with flows of different concentrations of ZOL. The response unit values were proportional to the ZOL concentration within the selected ranges **(B)** Calibration curve of the steady-state binding response against the ZOL concentrations.

As the ZOL in solution was injected and flows across a sensor chip surface fixed with RANKL, the continuous binding of ZOL and RANKL resulted in a change in the refraction angle and the mass on the sensor surface, which affected the response signal (Figure 3). Through the SPR experiment, we found that ZOL, with the K_D_ value of 2.28 × 10^−4^ M, could directly bind to the target RANKL protein. The resonance units (RU) values increased significantly with incremental increases in ZOL dose from 1.56 × 10^−5^ to 2.5 × 10^−4^ M, which indicated that ZOL directly bound with RANKL in a concentration-dependent manner.

### Receptor activator of nuclear factor-κB ligand trimer structure modelling and quality estimation

The original 3D structure of the RANKL trimer, as obtained by rotating symmetric polymeric docking, was optimized in a solvent to simulate the real physiological environment by performing a 30-ns MD simulation. We saved the structure in the stable state from the MD trajectory and assessed its structural quality (for detailed analysis of the modelling results and structural quality assessment, please see the Supplementary Material).

### Identification and analysis of binding site

**FIGURE 4:**
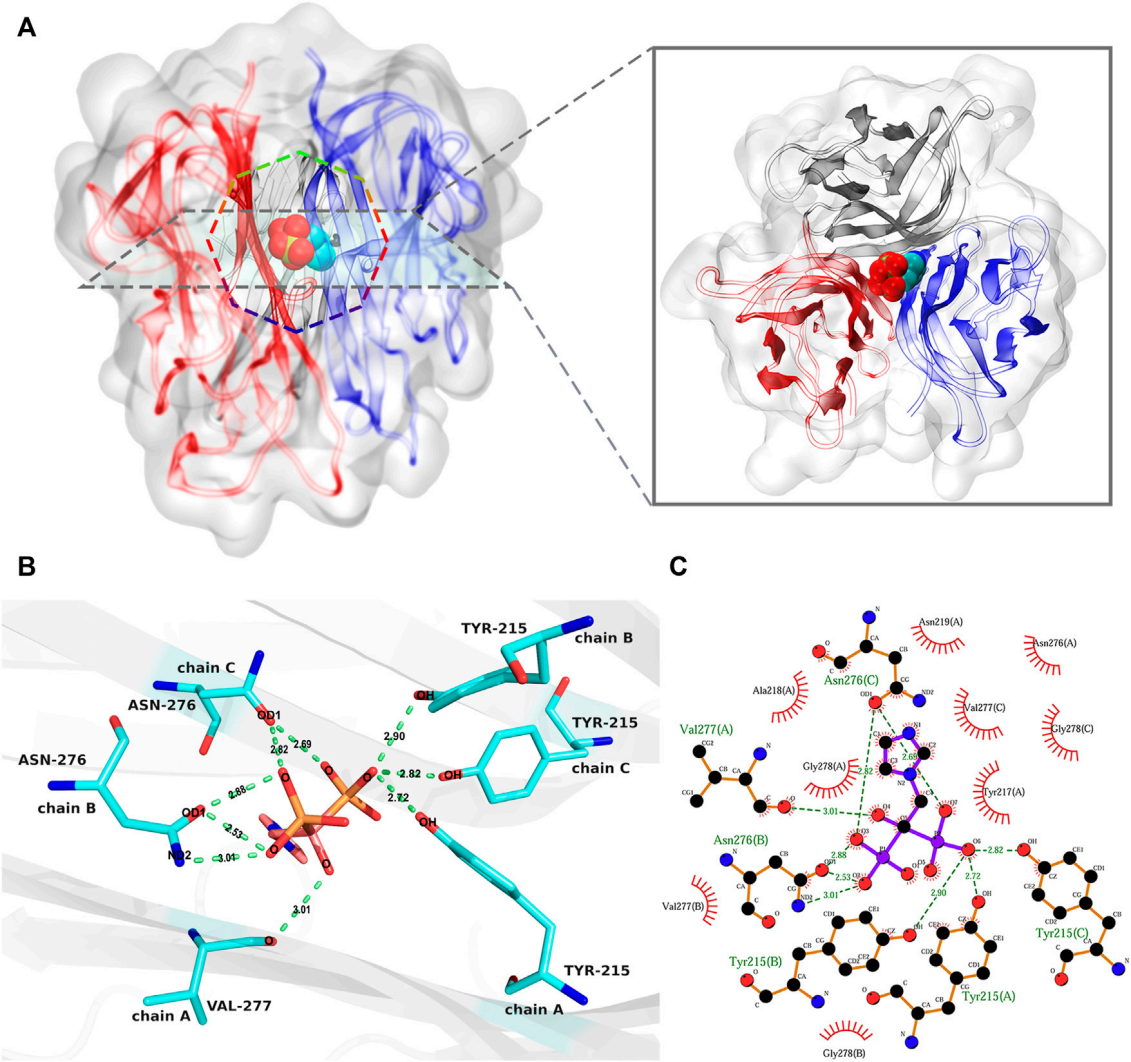
The analysis of the best binding pose of RANKL–ZOL complex. **(A)** shows the 3D conformation of the RANKL–ZOL complex from the front and vertical views, the structure of the RANKL trimer is shown as cartoon representation, and the structure of ZOL is shown as sphere representation. **(B,C)** depict the detailed interaction of RANKL and ZOL; **(B)** shows a 3D view of the hydrogen-bonding interactions between ZOL and RANKL; **(C)** shows the 2D interaction profile of RANKL–ZOL, in which the red lashes represent the residues engaged in hydrophobic interactions; the green dotted lines represent the hydrogen bonds.

Molecular docking is a process of thermodynamic equilibrium; the binding conformation with the lowest affinity energy is regarded as stable (Trott and Olson, 2010; Ferreira et al., 2015). Binding affinity is an important indicator for evaluating the strength of stable binding between a small molecule and a target protein. In this study, the lowest binding affinity of the docking simulations was −6.8 kcal/mol. According to the corresponding binding conformation with the lowest binding affinity, ZOL bound to the channel of the RANKL trimer (Figure 4A). The diagram of protein–ligand interaction analysis shows that the main types of interaction are hydrogen bonding and hydrophobic interaction (Figures 4B,C; Supplementary Table S1). The hydroxyl group of Tyr215 in chains A, B, and C forms hydrogen bonds of 2.72, 2.90, and 2.82 Å with the O6 oxygen atom of ZOL, respectively, and the oxygen atom of Val277 (A) forms a hydrogen bond of 3.01 Å with the O4 oxygen atom of ZOL. The carboxyl oxygen atom OD1 of Asn276 (B) forms two hydrogen bonds with the O3 and O2 oxygen atoms of ZOL, both lengths are 2.88 and 2.53 Å, respectively. In addition, the side-chain nitrogen atom ND2 of Asn276 (B) forms a hydrogen bond of 3.01 Å with the O2 oxygen atom of ZOL. The carboxyl oxygen atom OD1 in Asn276 (C) forms 2.82 and 2.69 Å hydrogen bonds with the O3 and O7 oxygen atoms in ZOL, respectively. In addition, Tyr217, Ala218, Asn219, Asn276, and Gly278 of chain A, Val277 and Gly278 of chain B, and Val277 and Gly278 of chain C constitute the hydrophobic region of the binding site. The analysis of other potential binding conformations was illustrated in the Supplementary Material.

### Molecular dynamics study of zoledronic acid and receptor activator of nuclear factor-κB ligand complex

**FIGURE 5:**
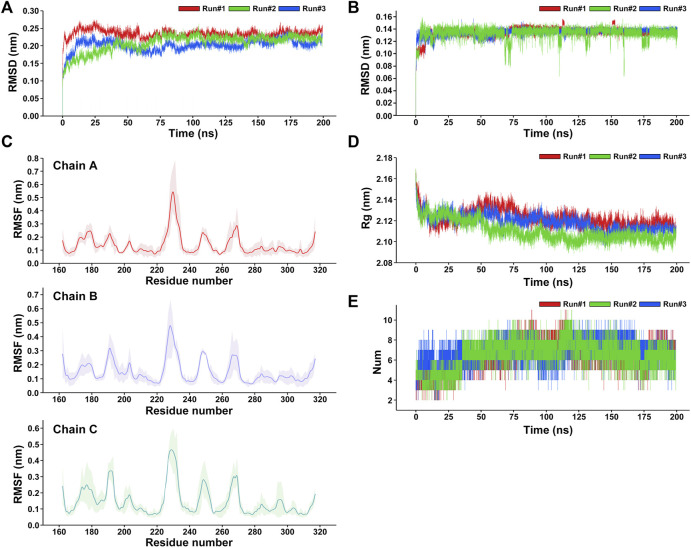
Analysis of the three repeated 200-ns molecular dynamics simulations of the RANKL–ZOL docking complex. **(A)** RMSD plot of the receptor protein RANKL for three repeated MD simulations. **(B)** RMSD plot of the ligand ZOL for three repeated MD simulations. **(C)** RMSF plot of the receptor protein RANKL, showing that the residues with strong flexibility are concentrated in fragments 188–195, 225–230, 245–253, and 265–270; and the structures of residues in fragments 215–222, 275–315 are relatively stable in the MD simulation. The RMSF values are represented as mean ± SD, where the average RMSF of each residue for three repeated simulations is shown as a solid line, and the fluctuation range of the RMSF value of each residue on the curve is shown in light color. **(D)** Rg plot of the receptor protein RANKL with respect to time (ns) for three repeated MD simulations presenting the comparative compactness. **(E)** Profile of the dynamics of intermolecular hydrogen bonds within the complex, shows the time distribution of the number of hydrogen bonds between ZOL and RANKL in the MD simulation. During the period 0–36 ns, the number of hydrogen bonds between ZOL and RANKL is mainly concentrated between 3 and 6. During the period 36–200 ns, the number of hydrogen bonds increased to between 5 and 11.

After the three repeated 200-ns MD simulations of the RANKL–ZOL binding complex, the root-mean-square deviation (RMSD) values of the protein backbone and ligand ZOL in the simulation system were respectively calculated to observe their structural stability during the MD simulation (Gulzar et al., 2019). RMSD is an important indicator reflecting the stability of the structure. The results show that the fluctuations of RMSD of RANKL are similar in three repeated simulations (Figure 5A). In the early 0–12 ns stage of three repeated simulations, the RANKL structure in aqueous solution fluctuated obviously, the RMSD curves of Run 1, Run 2 and Run 3 in this phase showed a rapidly upward trend. We speculated that this was because in the previous docking process, the receptor protein RANKL was set as rigid and the ligand ZOL structure was flexible. After the restraint released in the initial stage of the MD simulation, the protein structure was constantly being adjusted, and the RMSD thus had large fluctuations so that the conformations of ZOL and RANKL could be induced to fit each other and achieve an accurate binding pose. In addition, the solvation of the system also promoted the dynamic movement of the RANKL–ZOL complex structure, resulting in structural changes at the initial stage. During the period of 12–100 ns, the fluctuations of RMSD curves for three repeated simulations gradually became smaller and converge. Among the three replicates, the RMSD of RANKL in Run 2 showed a gradual increase in the period of 12–75 ns and reached the convergence after 75 ns, the values stabilized at the level of 0.225 nm. Meanwhile, the RMSD fluctuations of Run 1 and Run 2 slightly decreased and converged at 80 ns From 100 to 200 ns of three repeated simulations, the RMSD curves showed good stability, indicating that the receptor protein structures in three replicates were relatively stable and reached convergence. Compared to the receptor protein, the ligand ZOL rapidly reached convergence in the three repeated simulations. The RMSD curves were relatively stable in the simulations (Figure 5B). The structure of the ZOL ligand sharply fluctuated in the 0–20 ns period of the three repeated simulations. Among them, the RMSD of ZOL in Run 1 rapidly increased and stabilized temporarily at 0.11 nm during the first 8 ns, then the values reached convergence following the fluctuations in the period of 12–18 ns. For the RMSD of ZOL in Run 2, the curve rose and temporarily stayed at the level of 0.1 nm in 0–3 ns, then, in the following 4 ns, the RMSD increased to 0.16 nm. In the period of 7–20 ns, the RMSD first gradually decreased from 0.16 to 0.12 nm, then rose and stably fluctuated around 0.135 nm. Regarding the Run 3, the RMSD curve of ZOL showed the obvious increase during the period of 0–20 ns and the RMSD values stably fluctuated at 0.135 nm. While there existed a small fluctuation at 16 ns, ZOL in Run 3 was relatively stable at the beginning of the simulation compared with the other 2 replicates. Based on the three repeated simulations, we could observe that the ligand ZOL structure was in the state of continuous adjusting during the first 20 ns of the simulations. After this, atomic fluctuations in the ZOL conformation did not change much until the end of the simulation. Despite small fluctuations appeared in the period 116–117 and 153–156 ns of Run 1 and 66–75, 108–114, and 170–178 ns of Run 2, the overall structure was relatively stable, indicating that the ligand ZOL was able to stably bind to RANKL.

**FIGURE 6:**
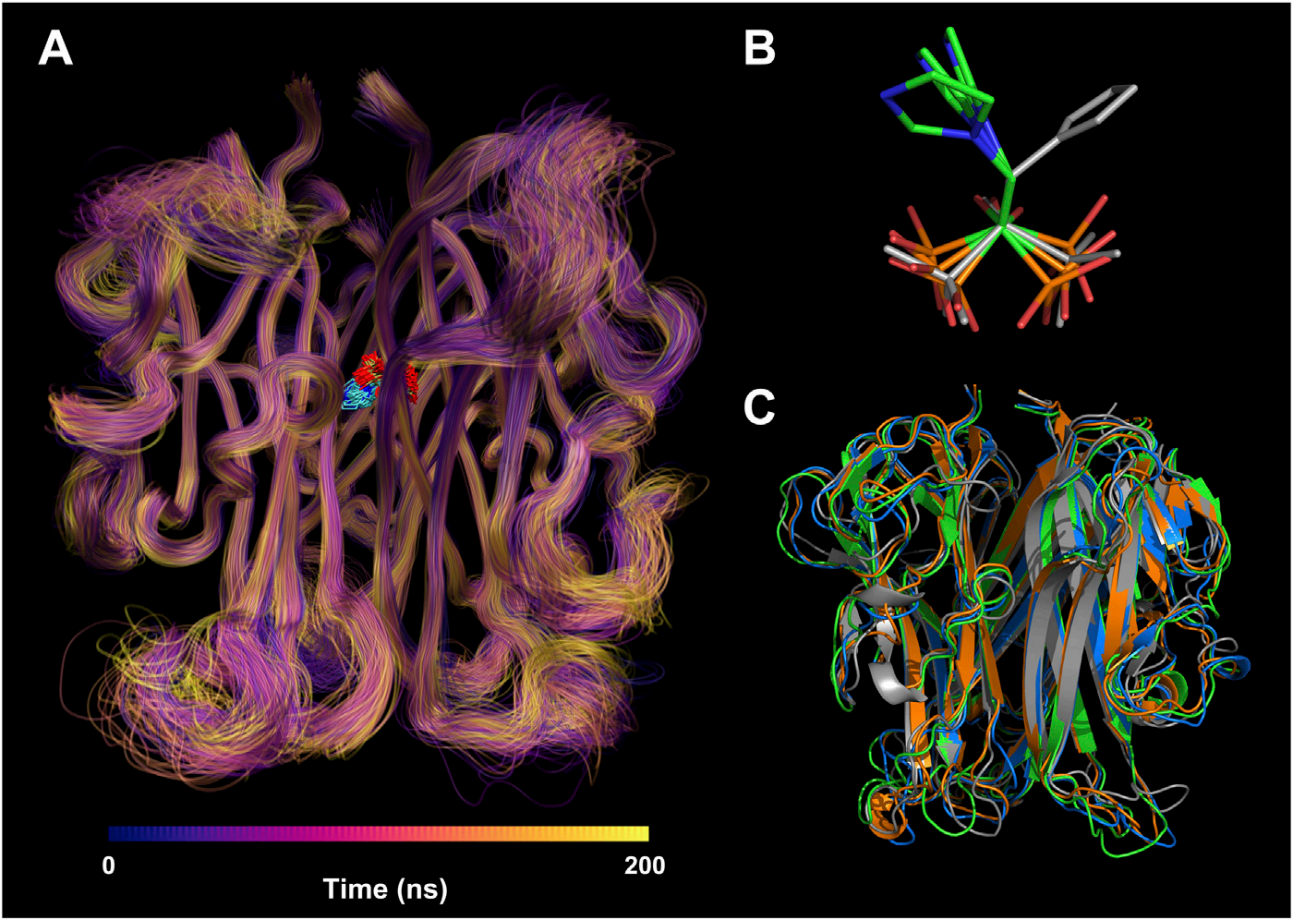
Structural visualization of RANKL-ZOL complex in molecular dynamics simulation. **(A)** Visualization of the MD trajectory of the receptor RANKL in Run 1. The trajectories are colored from deep blue to yellow according to their chronological order; the regions with concentrated trajectories indicate that the structural movement is relatively stable; conversely, regions with loose trajectories imply high structural flexibility. The trajectory superposition of the ligand ZOL is illustrated at the binding site. **(B)** The conformational changes of ZOL structures before and after the MD simulation, where the gray stick structure represents the initial conformation before MD simulation, and the colored stick structures represent the resulting conformations after three repeated MD simulations. **(C)** The conformational changes of RANKL structures before and after the MD simulation, where the gray cartoon structure represents the initial conformation before MD simulation, and the colored cartoon structures represent the resulting conformations after three repeated MD simulations.

To understand the per-residue flexibility in the MD simulation, we calculated the root-mean-square fluctuation (RMSF) for the individual residues of RANKL (Figure 5C). According to Run 1, Run 2, and Run 3, it was found that the residue flexibility trend of each chain of RANKL was similar, and the residues with strong flexibility were mainly distributed in the loop region of the side chain; these were concentrated in residue fragments 188–195, 225–230, 245–253, and 265–270. Furthermore, the structures of the residues in fragments 215–222, 275–315 were relatively stable in the MD simulation, which contributed to the stable binding of RANKL and ZOL. Given that the RMSF fluctuations for the three replicates were similar, we superimposed the trajectories of Run 1 to visualize the structural movement and intuitively displayed the structural fluctuations of the RANKL backbone, which was helpful for vividly understanding the RMSF results (Figure 6A). We found that there were little structural changes in the ligand-binding regions inside the receptor protein before and after the simulation, while larger structural movements occurred in the peripheral loop regions and the structure connections, which was consistent with the RMSF results analysis. Moreover, the structural fluctuations of the ligand ZOL were mainly concentrated in the imidazole ring, where the imidazole ring showed a large angle rotation after MD simulation. At the same time, the P-C-P structure of ZOL showed the high stability, indicating that the P-C-P nucleus, acting as a bone hook, was contributed to anchor the drug to RANKL (Figures 6B,C).

Additionally, we evaluated the structural stability and fold–stretch dynamic behavior of the receptor protein RANKL by calculating its radius of gyration (Rg) of three repeated simulations (Ivankov et al., 2009) (Figure 5D). In the three replicates, we found the similar trends in the Rg changes of RANKL. During 0–15 ns of three repeated simulations, the RANKL structure showed a significant folding trend, and the average Rg value at this stage decreased obviously from 2.17 to 2.12 nm, indicating that the compactness of the structure increased. Then, during 15–50 ns, the Rg values of Run 2 and Run 3 showed the similar changes, fluctuating at the level of 2.12 nm, while the Rg values of Run 1 showed an increasing trend from 2.12 to 2.14 nm. From the three replicates, we observed that the structure of receptor was still in instability in the period of 15–50 ns. During the period 50–200 ns, the Rg values gradually decreased and stabilized at the level of 2.117 nm in all three replicates, implying that the overall structure of RANKL remained in a tight and stably folded state in this period.

Hydrogen-bonding interactions play a key role in the stable binding of the RANKL–ZOL complex, and they are of great significance for us in the exploration of the interactions between the molecules (Tanveer et al., 2021). We analyzed the time distribution of the number of hydrogen bonds between ZOL and RANKL in the three repeats of MD simulations, the results are shown in Figure 5E. We found similar trends in the number of hydrogen bonds in the three repeated simulations, where the number of hydrogen bonds ranged from 2–11 and the average number of hydrogen bond was 6.55 ± 1. During the period from 0 to 36 ns, the number of intermolecular hydrogen bonds was mainly concentrated between 3 and 7, where there were 3–6 hydrogen bonds between RANKL and ZOL in Run 1 and Run 2, and 4–7 intermolecular hydrogen bonds in Run 3. However, the number of hydrogen bonds increased and fluctuated stably at the range of 5–11 from 36 to 170 ns in this period, the number of hydrogen bonds in Run 3 temporarily dropped to about 6 at 100 ns and then increased to 6–9, while the number of hydrogen bonds in Run 1 and Run 2 were relatively stable. In the last 30 ns of simulations, fluctuation range of the number of hydrogen bonds decreased and stabilized in 3–9 in all three repeated simulations. In contrast to the number of hydrogen bonds in the initial structure, the increases in the number of hydrogen bonds during the simulations may be ascribed to the fact that with the continuous structural relaxation of the RANKL–ZOL complex, the positions of the atoms in the binding region move, which leads potential intermolecular hydrogen bonds to be exposed in the following simulation process. (A movie of the 200-ns MD simulation was available in the Supplementary Material.)

### Binding free energy calculation and per-residue free energy decomposition

We extracted RANKL–ZOL complex structure snapshots from the last 50 ns of the three repeated MD trajectories of the stable system, and we calculated the binding free energy between ZOL and RANKL using the MM-PBSA method (Supplementary Table S2). The average calculated binding free energy was −70.67 ± 2.62 kJ/mol, indicating that the interaction between ZOL and RANKL is spontaneous and that they fit well together. The energy contribution values of the intermolecular van der Waals interactions, electrostatic interactions, and non-polar solvation were all less than zero, which promotes the process of targeted binding of ZOL with RANKL and the formation of a stable docking conformation. The average values of these interactions were −138.14, −123.50, and −13.07 kJ/mol, respectively. It is noteworthy that the van der Waals and electrostatic interactions have decisive effects on the stable binding of RANKL and ZOL. Conversely, the polar solvation interaction is not conducive to binding: the average free-energy value is 204.04 kJ/mol, which is greater than zero.

**FIGURE 7:**
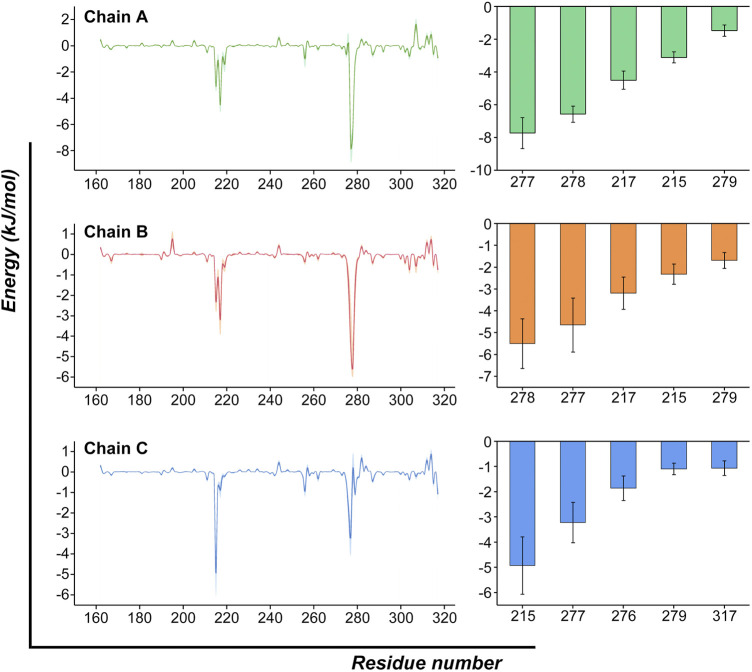
Per-residue binding free-energy calculation. Per-residue binding free-energy contribution spectra of the interaction between RANKL and ZOL. The bar graph on the right lists the top-five residues of each chain and shows their energy contributions. (The results are presented as Mean ± SD).

We then decomposed the overall binding free energy to the per-residue level to further explore the contribution each residue to the interaction between ZOL and RANKL (Figure 7; Supplementary Table S3). The residues with high energy contributions were mainly located in residue ranges 215–220 and 275–280; these residues were contributive to the binding of RANKL–ZOL, and they further confirmed the binding site predicted by the previous molecular docking simulations. The residues with binding free-energy values less than −3.5 kJ/mol included Tyr217 (A), Val277 (A), Gly278 (A), Val277 (B), Gly278 (B), and Tyr215 (C), and their binding energies were −4.49, −7.72, −6.56 −4.65, −5.50, and −4.93 kJ/mol, respectively. All of these key residues of the binding region were found to contribute significantly to the interaction between ZOL and RANKL, and this is the basis for ZOL to interact with RANKL.

## Discussion

In this study, we first demonstrated the binding interactions between ZOL and RANKL and found that hydrogen bonding and hydrophobic effects were the main types of binding interaction, and disclosed the corresponding residues. Additionally, the process of the three repeated 200-ns MD simulations confirmed the stability of the binding conformation and the reliability of the docking results. We then calculated the binding free energy of RANKL–ZOL and identified the key residues of the binding sites. In addition to the binding of crystal structures of complexes between ZOL and FPPS, our results provide a basis for exploring the biological effects of ZOL.

BRONJ is a rare, but potentially serious, adverse event associated with high cumulative doses of antiresorptives (Li et al., 2021). ZOL is the most widely used antiresorptive agent, and it has the function of inhibiting osteoclast maturation and differentiation. It acts by binding the mineral component of bone and decreasing RANKL expression in human osteoblasts (Pan et al., 2004; Watanabe et al., 2020). The inhibition of RANKL leads to the loss of osteoclasts from bone surfaces, and reducing bone remodeling (Nagaoka et al., 2015). According to the calculated kinetic parameters, we observed that ZOL remarkably decreased MS/BS, MAR and BFR/BS at 6–8 and 8–10 weeks, diminishing bone remodeling and turnover in ZOL-treated rats compared to the control, as was in accordance with previous report (Allen, 2009). The administration of RANKL in experimental rats could alleviate the ZOL-induced decrease in bone turnover and reduce the occurrence of osteonecrosis (Lloyd et al., 2008; Allen, 2009).

ZOL-induced BRONJ has multiple causes, including inhibition of angiogenesis, influence of bone turnover, genetic predisposition, etc. Among them, RANKL-mediated bone turnover is important in the development of BRONJ (Parfitt et al., 1987; Watanabe et al., 2020). Researchers have reported that RANKL plays an essential role in the development of osteoclasts. The extracellular portion of RANKL is cleaved proteolytically to produce soluble trimeric RANKL, which is a crucial process that down-regulates local osteoclastogenesis (Hikita et al., 2006). A number of experiments have demonstrated that RANKL stimulates the differentiation, survival, and fusion of osteoclastic precursor cells, and it activates mature osteoclasts and prolongs their lifespan by inhibiting apoptosis. Consistent with these effects, administration of RANKL to mice *in vivo* resulted in severe osteoporosis associated with increased osteoclastic activity (Jimi et al., 1999; Gifre et al., 2017). Based on this theory, we emphasize the interaction mechanism between ZOL and protein RANKL from the perspectives in tissue, molecule and structure to reveal the pathogenic basis of ZOL suppressing bone turnover and thus inducing BRONJ.

ZOL markedly decreases RANKL expression, however, this is not related to a significant change in the RANKL gene, indicating that ZOL may regulate RANKL expression post-translationally (Pan et al., 2004). Additionally, ZOL strongly inhibits RANKL-induced upregulation of RANK in a dose-dependent manner, which is likely to be associated with the suppression of the NF-κB pathway (Baron et al., 2011). Nevertheless, the underlying mechanism of the interaction between ZOL and RANKL remains unknown, and the structure basis of this interaction needs to be further investigated.

In order to further explore the molecular mechanism of ZOL action, we found that ZOL could bind to RANKL using SPR experiment. The interaction between ZOL and RANKL belongs to the type of small molecule-protein interaction. In the present study, the detected equilibrium dissociation constant was in the range of protein-small molecule binding and showed the moderate strength of intermolecular binding between ZOL and RANKL, which was similar to the interaction between RANKL and Pheophorbide A (K_D_ = 40 μM), of the same order of magnitude (Chypre et al., 2017; Porkolab et al., 2020). Furthermore, we detected that the binding interaction operated in a concentration-dependent manner. ZOL deposited in bone tissue will inhibit RANKL for a long time, and it not only inhibits mature osteoclasts but also prevents osteoclast precursors from differentiating and migrating towards inflammatory osteolysis lesions (Kimachi et al., 2011).

Molecular simulations can be used to mimic the interaction between a small molecule and a protein, and this has become an increasingly key tool for exploring pharmacological mechanisms (McConkey et al., 2003; Meng et al., 2011). To further explore the interaction between ZOL and RANKL, we modeled the structure of the human RANKL trimer and applied it in molecular docking simulations. Based on the binding conformations of the RANKL–ZOL complex, we monitored the binding site and validated that hydrogen bonding and hydrophobic interactions are effective. It is these interactions that might make ZOL exert its function of inhibiting RANKL and osteoclast differentiation.

We then performed three repeated 200-ns MD simulations, and this revealed the reliability and stability of the combination of the two molecules. The RMSD results showed that protein and ligand could quickly converge during the simulation, indicating that the structure of RANKL-ZOL complex were relatively stable. According to RMSF analysis, the residue regions with strong flexibility were mainly distributed in the side-chain loop regions, including residue fragments 188–195, 225–230, 245–253, and 265–270 of the three chains. Meanwhile, the structures of residue fragments 215–222, 275–315 had little conformational changes, which might contribute to the stable binding of ZOL and RANKL. The Rg assay also validated the structural stability and folding-dynamic behavior of RANKL binding with ZOL. Moreover, the continuous relaxation of the conformation of the RANKL–ZOL complex in the MD simulation led to an increased number of intermolecular hydrogen bonds, which is conducive to the RANKL–ZOL binding. The average binding free energy between ZOL and RANKL was found to be −70.67 kJ/mol, and van der Waals forces and electrostatic interactions contribute significantly to stable binding between the molecules. The residues with the most prominent contributions to the combination were Tyr217 (A), Val277 (A), Gly278 (A), Val277 (B), Gly278 (B), and Tyr215 (C).

In the current study, we found that ZOL was able to stably bind within the RANKL trimer channel and assumed that ZOL affected the normal biological functions of RANKL *in vivo* by interfering with the hydrophobic kernel of the trimer according to the structure features. Therefore, we can design the targeted molecules based on the ligand binding site to reduce osteonecrosis by competitively occupying the original binding site with ZOL and stabilizing the trimerization of RANKL (Ostrem et al., 2013; De Vita et al., 2019). Moreover, our data provide structure-based investigation of ZOL binding site on RANKL that helps to instruct us in the further studies on key residues.

In summary, we demonstrated the interaction between ZOL and RANKL, identified the key residues of the binding sites, and calculated that the binding free energy between ZOL and RANKL. Our results reveal the mechanism of bisphosphonate and are also conducive to shed light on the mechanisms of BRONJ occurrence, which provide a safe and effective basis for bisphosphonate treatment of osteoporosis that is widespread in the population.

## Data Availability

The original contributions presented in the study are included in the article/Supplementary Materials, further inquiries can be directed to the corresponding authors.

## References

[B1] Aguirre J. I. Castillo E. J. Kimmel D. B. (2021). Preclinical models of medication-related osteonecrosis of the jaw (MRONJ). Bone 153, 116184. 10.1016/j.bone.2021.116184 34520898 PMC8743993

[B2] Allen M. R. (2009). Bisphosphonates and osteonecrosis of the jaw: Moving from the bedside to the bench. Cells Tissues Organs 189 (1-4), 289–294. 10.1159/000151371 18698128 PMC2824205

[B3] Baron R. Ferrari S. Russell R. G. (2011). Denosumab and bisphosphonates: Different mechanisms of action and effects. Bone 48 (4), 677–692. 10.1016/j.bone.2010.11.020 21145999

[B4] Chypre M. Madel M. B. Chaloin O. Blin-Wakkach C. Morice C. Mueller C. G. (2017). Porphyrin derivatives inhibit the interaction between receptor activator of NF-κB and its ligand. ChemMedChem 12 (20), 1697–1702. 10.1002/cmdc.201700462 28885764

[B5] De Vita S. Lauro G. Ruggiero D. Terracciano S. Riccio R. Bifulco G. (2019). Protein preparation automatic protocol for high-throughput inverse virtual screening: Accelerating the target identification by computational methods. J. Chem. Inf. Model. 59 (11), 4678–4690. 10.1021/acs.jcim.9b00428 31593460

[B6] Dunford J. E. Thompson K. Coxon F. P. Luckman S. P. Hahn F. M. Poulter C. D. (2001). Structure-activity relationships for inhibition of farnesyl diphosphate synthase *in vitro* and inhibition of bone resorption *in vivo* by nitrogen-containing bisphosphonates. J. Pharmacol. Exp. Ther. 296 (2), 235–242. 11160603

[B7] El Bakri Y. Anouar E. H. Ahmad S. Nassar A. A. Taha M. L. Mague J. T. (2021). Synthesis and identification of novel potential molecules against COVID-19 main protease through structure-guided virtual screening approach. Appl. Biochem. Biotechnol. 193, 3602–3623. 10.1007/s12010-021-03615-8 34324152 PMC8319192

[B8] Febres-Molina C. Aguilar-Pineda J. A. Gamero-Begazo P. L. Barazorda-Ccahuana H. L. Valencia D. E. Vera-Lopez K. J. (2021). Structural and energetic affinity of annocatacin B with ND1 subunit of the human mitochondrial respiratory complex I as a potential inhibitor: An in silico comparison study with the known inhibitor rotenone. Polym. (Basel) 13 (11), 1840. 10.3390/polym13111840 PMC8199665 34199390

[B9] Ferreira L. G. Dos Santos R. N. Oliva G. Andricopulo A. D. (2015). Molecular docking and structure-based drug design strategies. Molecules 20 (7), 13384–13421. 10.3390/molecules200713384 26205061 PMC6332083

[B10] Gifre L. Ruiz-Gaspà S. Carrasco J. L. Portell E. Vidal J. Muxi A. (2017). Effect of recent spinal cord injury on the OPG/RANKL system and its relationship with bone loss and the response to denosumab therapy. Osteoporos. Int. 28 (9), 2707–2715. 10.1007/s00198-017-4090-4 28580511

[B11] Giusti A. Bianchi G. (2015). Treatment of complex regional pain syndrome type I with bisphosphonates. RMD Open 1 (1), e000056. 10.1136/rmdopen-2015-000056 26557377 PMC4632140

[B12] Gulzar M. Ali S. Khan F. I. Khan P. Taneja P. Hassan M. I. (2019). Binding mechanism of caffeic acid and simvastatin to the integrin linked kinase for therapeutic implications: A comparative docking and MD simulation studies. J. Biomol. Struct. Dyn. 37 (16), 4327–4337. 10.1080/07391102.2018.1546621 30488773

[B13] Hikita A. Yana I. Wakeyama H. Nakamura M. Kadono Y. Oshima Y. (2006). Negative regulation of osteoclastogenesis by ectodomain shedding of receptor activator of NF-kappaB ligand. J. Biol. Chem. 281 (48), 36846–36855. 10.1074/jbc.M606656200 17018528

[B14] Huang X. L. Huang L. Y. Cheng Y. T. Li F. Zhou Q. Wu C. (2019). Zoledronic acid inhibits osteoclast differentiation and function through the regulation of NF-κB and JNK signalling pathways. Int. J. Mol. Med. 44 (2), 582–592. 10.3892/ijmm.2019.4207 31173157 PMC6605660

[B15] Ivankov D. N. Bogatyreva N. S. Lobanov M. Y. Galzitskaya O. V. (2009). Coupling between properties of the protein shape and the rate of protein folding. PLoS One 4 (8), e6476. 10.1371/journal.pone.0006476 19649298 PMC2714458

[B16] Jimi E. Akiyama S. Tsurukai T. Okahashi N. Kobayashi K. Udagawa N. (1999). Osteoclast differentiation factor acts as a multifunctional regulator in murine osteoclast differentiation and function. J. Immunol. 163 (1), 434–442. 10384146

[B17] Jones D. H. Kong Y. Y. Penninger J. M. (2002). Role of RANKL and RANK in bone loss and arthritis. Ann. Rheum. Dis. 61 (2), ii32–9. 10.1136/ard.61.suppl_2.ii32 12379618 PMC1766717

[B18] Khan A. A. Morrison A. Hanley D. A. Felsenberg D. McCauley L. K. O'Ryan F. (2015). Diagnosis and management of osteonecrosis of the jaw: A systematic review and international consensus. J. Bone Min. Res. 30 (1), 3–23. 10.1002/jbmr.2405 25414052

[B19] Kimachi K. Kajiya H. Nakayama S. Ikebe T. Okabe K. (2011). Zoledronic acid inhibits RANK expression and migration of osteoclast precursors during osteoclastogenesis. Naunyn. Schmiedeb. Arch. Pharmacol. 383 (3), 297–308. 10.1007/s00210-010-0596-4 21225243

[B20] Kumar A. Sharma M. Richardson C. D. Kelvin D. J. (2022). Potential of natural alkaloids from jadwar (delphinium denudatum) as inhibitors against main protease of COVID-19: A molecular modeling approach. Front. Mol. Biosci. 9, 898874. 10.3389/fmolb.2022.898874 35620478 PMC9127362

[B21] Lam J. Nelson C. A. Ross F. P. Teitelbaum S. L. Fremont D. H. (2001). Crystal structure of the TRANCE/RANKL cytokine reveals determinants of receptor-ligand specificity. J. Clin. Invest. 108 (7), 971–979. 10.1172/JCI13890 11581298 PMC200957

[B22] Leibbrandt A. Penninger J. M. (2008). RANK/RANKL: Regulators of immune responses and bone physiology. Ann. N. Y. Acad. Sci. 1143, 123–150. 10.1196/annals.1443.016 19076348

[B23] Li J. Sarosi I. Yan X. Q. Morony S. Capparelli C. Tan H. L. (2000). RANK is the intrinsic hematopoietic cell surface receptor that controls osteoclastogenesis and regulation of bone mass and calcium metabolism. Proc. Natl. Acad. Sci. U. S. A. 97 (4), 1566–1571. 10.1073/pnas.97.4.1566 10677500 PMC26475

[B24] Li M. Wang J. Yu Y. Zhou Y. Shi Y. Zhang W. (2021). Characterization of mesenchymal stem cells derived from bisphosphonate-related osteonecrosis of the jaw patients' gingiva. Stem Cell Rev. Rep. 18, 378–394. 10.1007/s12015-021-10241-8 34553308 PMC8799576

[B25] Liu H. Wang S. H. Chen S. C. Chen C. Y. Lo J. L. Lin T. M. (2016). Immune modulation of CD4+CD25+ regulatory T cells by zoledronic acid. BMC Immunol. 17 (1), 45. 10.1186/s12865-016-0183-7 27887569 PMC5124310

[B26] Lloyd S. A. Yuan Y. Y. Kostenuik P. J. Ominsky M. S. Lau A. G. Morony S. (2008). Soluble RANKL induces high bone turnover and decreases bone volume, density, and strength in mice. Calcif. Tissue Int. 82 (5), 361–372. 10.1007/s00223-008-9133-6 18465074

[B27] Luan X. Lu Q. Jiang Y. Zhang S. Wang Q. Yuan H. (2012). Crystal structure of human RANKL complexed with its decoy receptor osteoprotegerin. J. Immunol. 189 (1), 245–252. 10.4049/jimmunol.1103387 22664871

[B28] McConkey B. J. Sobolev V. Edelman M. (2003). Discrimination of native protein structures using atom-atom contact scoring. Proc. Natl. Acad. Sci. U. S. A. 100 (6), 3215–3220. 10.1073/pnas.0535768100 12631702 PMC152272

[B29] Meng X. Y. Zhang H. X. Mezei M. Cui M. (2011). Molecular docking: A powerful approach for structure-based drug discovery. Curr. Comput. Aided. Drug Des. 7 (2), 146–157. 10.2174/157340911795677602 21534921 PMC3151162

[B30] Munasinghe A. Lin P. Colina C. M. (2017). Unraveling binding interactions between human RANKL and its decoy receptor osteoprotegerin. J. Phys. Chem. B 121 (39), 9141–9148. 10.1021/acs.jpcb.7b06687 28945380

[B31] Nagaoka Y. Kajiya H. Ozeki S. Ikebe T. Okabe K. (2015). Mevalonates restore zoledronic acid-induced osteoclastogenesis inhibition. J. Dent. Res. 94 (4), 594–601. 10.1177/0022034514564187 25535203

[B32] Nagar P. R. Gajjar N. D. Dhameliya T. M. (2021). In search of SARS CoV-2 replication inhibitors: Virtual screening, molecular dynamics simulations and ADMET analysis. J. Mol. Struct. 1246, 131190. 10.1016/j.molstruc.2021.131190 34334813 PMC8313085

[B33] Nancollas G. H. Tang R. Phipps R. J. Henneman Z. Gulde S. Wu W. (2006). Novel insights into actions of bisphosphonates on bone: Differences in interactions with hydroxyapatite. Bone 38 (5), 617–627. 10.1016/j.bone.2005.05.003 16046206

[B34] Nieto A. Colilla M. Balas F. Vallet-Regí M. (2010). Surface electrochemistry of mesoporous silicas as a key factor in the design of tailored delivery devices. Langmuir 26 (7), 5038–5049. 10.1021/la904820k 20222698

[B35] Ogunyemi O. M. Gyebi G. A. Saheed A. Paul J. Nwaneri-Chidozie V. Olorundare O. (2022). Inhibition mechanism of alpha-amylase, a diabetes target, by a steroidal pregnane and pregnane glycosides derived from Gongronema latifolium Benth. Front. Mol. Biosci. 9, 866719. 10.3389/fmolb.2022.866719 36032689 PMC9399641

[B36] Ohno K. Mori K. Orita M. Takeuchi M. (2011). Computational insights into binding of bisphosphates to farnesyl pyrophosphate synthase. Curr. Med. Chem. 18 (2), 220–233. 10.2174/092986711794088335 21110804 PMC3343387

[B37] Ostrem J. M. Peters U. Sos M. L. Wells J. A. ShokatK-Ras K. M. (2013). K-Ras(G12C) inhibitors allosterically control GTP affinity and effector interactions. Nature 503 (7477), 548–551. 10.1038/nature12796 24256730 PMC4274051

[B38] Pan B. Farrugia A. N. To L. B. Findlay D. M. Green J. Lynch K. (2004). The nitrogen-containing bisphosphonate, zoledronic acid, influences RANKL expression in human osteoblast-like cells by activating TNF-alpha converting enzyme (TACE). J. Bone Min. Res. 19 (1), 147–154. 10.1359/jbmr.2004.19.1.147 14753746

[B39] Parfitt A. M. Drezner M. K. Glorieux F. H. Kanis J. A. MallucHe H. Meunier P. J. (1987). Bone histomorphometry: Standardization of nomenclature, symbols, and units. Report of the ASBMR histomorphometry nomenclature committee. J. Bone Min. Res. 2 (6), 595–610. 10.1002/jbmr.5650020617 3455637

[B40] Porkolab V. Pifferi C. Sutkeviciute I. Ordanini S. Taouai M. Thepaut M. (2020). Development of C-type lectin-oriented surfaces for high avidity glycoconjugates: Towards mimicking multivalent interactions on the cell surface. Org. Biomol. Chem. 18 (25), 4763–4772. 10.1039/d0ob00781a 32608454

[B41] Rastelli G. Del Rio A. Degliesposti G. Sgobba M. (2010). Fast and accurate predictions of binding free energies using MM-PBSA and MM-GBSA. J. Comput. Chem. 31 (4), 797–810. 10.1002/jcc.21372 19569205

[B42] Schmid N. Eichenberger A. P. Choutko A. Riniker S. Winger M. Mark A. E. (2011). Definition and testing of the GROMOS force-field versions 54A7 and 54B7. Eur. Biophys. J. 40 (7), 843–856. 10.1007/s00249-011-0700-9 21533652

[B43] Soundia A. Hadaya D. Chau Y. Gkouveris I. Bezouglaia O. Dry S. (2021). Local RANKL delivery improves socket healing in bisphosphonate treated rats. Bone 148, 115945. 10.1016/j.bone.2021.115945 33836308 PMC9396533

[B44] Stachnik A. Yuen T. Iqbal J. Sgobba M. Gupta Y. Lu P. (2014). Repurposing of bisphosphonates for the prevention and therapy of nonsmall cell lung and breast cancer. Proc. Natl. Acad. Sci. U. S. A. 111 (50), 17995–18000. 10.1073/pnas.1421422111 25453078 PMC4273392

[B45] Stopeck A. T. Lipton A. Body J. J. Steger G. G. Tonkin K. de Boer R. H. (2010). Denosumab compared with zoledronic acid for the treatment of bone metastases in patients with advanced breast cancer: A randomized, double-blind study. J. Clin. Oncol. 28 (35), 5132–5139. 10.1200/JCO.2010.29.7101 21060033

[B46] Stroet M. Caron B. Visscher K. M. Geerke D. P. Malde A. K. Mark A. E. (2018). Automated topology builder version 3.0: Prediction of solvation free enthalpies in water and hexane. J. Chem. Theory Comput. 14 (11), 5834–5845. 10.1021/acs.jctc.8b00768 30289710

[B47] Subramanian N. Schumann-Gillett A. Mark A. E. O'Mara M. L. (2019). Probing the pharmacological binding sites of P-glycoprotein using umbrella sampling simulations. J. Chem. Inf. Model. 59 (5), 2287–2298. 10.1021/acs.jcim.8b00624 30540465

[B48] Tanveer A. Wani N. A. A. MohammedAlanazi M. A. H. B. Azmat Ali Khan S. Z. Zargar S. (2021). Binding of colchicine and ascorbic acid (vitamin C) to bovine serum albumin: An *in-vitro* interaction study using multispectroscopic, molecular docking and molecular dynamics simulation study. J. Mol. Liq. 333, 117542. 10.1016/j.molliq.2021.117542 PMC7969832 33753950

[B49] Trang N. M. Kim E. N. Lee H. S. Jeong G. S. (2022). Effect on osteoclast differentiation and ER stress downregulation by amygdalin and RANKL binding interaction. Biomolecules 12 (2), 256. 10.3390/biom12020256 35204757 PMC8961616

[B50] Trott O. Olson A. J. (2010). AutoDock Vina: Improving the speed and accuracy of docking with a new scoring function, efficient optimization, and multithreading. J. Comput. Chem. 31 (2), 455–461. 10.1002/jcc.21334 19499576 PMC3041641

[B51] Wang R. J. Zhao Q. T. Yu Y. J. Zhou Y. Q. Wang S. Y. (2021). Molecular mechanism of zoledronic acid inhibiting angiogenesis by semi flexible binding with vascular endothelial growth factor conformation. Zhonghua Kou Qiang Yi Xue Za Zhi 56 (7), 679–686. 10.3760/cma.j.cn112144-20200729-00441 34275224

[B52] Wang S. Zhang Z. Xia L. Zhao J. Sun X. Zhang X. (2010). Systematic evaluation of a tissue-engineered bone for maxillary sinus augmentation in large animal canine model. Bone 46 (1), 91–100. 10.1016/j.bone.2009.09.008 19761881

[B53] Watanabe J. Sakai K. Urata Y. Toyama N. Nakamichi E. Hibi H. (2020). Extracellular vesicles of stem cells to prevent BRONJ. J. Dent. Res. 99 (5), 552–560. 10.1177/0022034520906793 32119600

[B54] Xiao Y. Karttunen M. Jalkanen J. Mussi M. C. M. Liao Y. Grohe B. (2015). Hydroxyapatite growth inhibition effect of pellicle statherin peptides. J. Dent. Res. 94 (8), 1106–1112. 10.1177/0022034515586769 26116492

[B55] Yan X. Wang Y. Meng T. Yan H. (2021). Computational insights into the influence of substitution groups on the inclusion complexation of β-cyclodextrin. Front. Chem. 9, 668400. 10.3389/fchem.2021.668400 34095084 PMC8176092

[B56] Yang G. Singh S. Chen Y. Hamadeh I. S. Langaee T. McDonough C. W. (2019). Pharmacogenomics of osteonecrosis of the jaw. Bone 124, 75–82. 10.1016/j.bone.2019.04.010 31022475

[B57] Yin F. Cao R. Goddard A. Zhang Y. Oldfield E. (2006). Enthalpy versus entropy-driven binding of bisphosphonates to farnesyl diphosphate synthase. J. Am. Chem. Soc. 128 (11), 3524–3525. 10.1021/ja0601639 16536518

[B58] Zhang N. Zhang Z. K. Yu Y. Zhuo Z. Zhang G. Zhang B. T. (2020). Pros and cons of denosumab treatment for osteoporosis and implication for RANKL aptamer therapy. Front. Cell Dev. Biol. 8, 325. 10.3389/fcell.2020.00325 32478071 PMC7240042

[B59] Zhou Y. Q. Son G. H. Shi Y. Q. Yu Y. J. Li M. Y. Zhang Q. (2021). Quantitative segmentation analysis of the radiological changes by using ITK-SNAP: Risk assessment of the severity and recurrence of medication-related osteonecrosis of the jaw. Int. J. Med. Sci. 18 (10), 2209–2216. 33859529 10.7150/ijms.56408 PMC8040413

