# Supplementary material for: Structural insights into the binding of zoledronic acid with RANKL via computational simulations
Source: Front Mol Biosci. 2022 Sep 19;9:992473. doi: 10.3389/fmolb.2022.992473 (PMC9527314; doi:10.3389/fmolb.2022.992473)
Supplement: Supplementary file 2 [file DataSheet1.docx]

**SUPPLEMENTAL MATERIAL**

*Detailed Method of Human RANKL Trimer Modelling*

In this study, we selected the structure of the soluble RANKL extracellular domain (residues 162 to 317) as the receptor protein^[1-3]^. The monomer structure of human RANKL (PDB ID:3URF, 2.70 Å) was obtained from the RCSB Protein Data Bank online database (<https://www.rcsb.org>), and the molecular visualization software PyMOL v2.3.4 was used to remove irrelevant structures, water molecules, and co-crystallized ligands. The monomer has a typical jellyroll-like β-sheet structure. (Fig. 1A) The monomer RANKL structure was submitted to the M-ZDOCK server (<https://zlab.umassmed.edu/m-zdock>) for rotating symmetric polymeric docking to construct the original structural model of the RANKL trimer (Fig. 1B).

In the structural modeling of proteins, MD simulation is usually used to improve original models ^[4]^. We used GROMACS v2019.3 to perform the MD simulation for the original trimer structure to eliminate possible stereo clashes and achieve a structure closer to the native state. The shape of the simulation box was set as cubic, and the GROMOS96 54A7 force field and the SPC water model were adopted. Then, six Na^+^ ions were added to the system to obtain electrostatic neutrality. The structure underwent 30 000 steps of energy minimization and was subjected to 100 ps NVT and 100 ps NPT equilibration phases. Subsequently, we conducted a 30-ns production MD simulation and selected the conformation in the stable state from the MD trajectory as the resulting conformation of the RANKL trimer and aligned it with the original structure (Fig. 1C, D). Finally, we estimated the quality of the optimized structure to ensure the accuracy of subsequent molecular docking. We submitted the optimized model to the RAMA online server (<https://zlab.umassmed.edu/bu/rama/>) to check the reliability of the dihedral angle (Φ, φ) in the amide plane of the residues and generate a Ramachandran plot^[5]^. In addition, the Verify3D program was applied to evaluate the compatibility between the three-dimensional structure of the model and its primary structure to check its quality^[6, 7]^.

**Results**

*Detailed analysis of RANKL Trimer Structure Modelling and Quality Estimation*

As described, the original RANKL trimer structure modeled by the online server was optimized through a 30-ns MD simulation. We choose the backbone as the original structure to perform least-squares fitting and calculate the RMSD of the target protein in the dynamic trajectory to check whether the protein structure was in a stable state. According to the RMSD plot, we found that the structure quickly reached a stable state in the process of the MD simulation (Supplementary Fig. 1). During the 0–8 ns period of the MD simulation, the RMSD values varied significantly, indicating that the protein structure fluctuated greatly during this stage. The RMSD values then fluctuated stably around 0.33 nm in the 8–30 ns period, implying that the RANKL trimer structure had converged.

We saved the structure from the last frame of the trajectory in the stable state as the resulting conformation of the RANKL trimer and estimated the structure quality. The Ramachandran plot suggested that 92.736% of the residues of this model were located in the highly preferred region, 6.538% were in the allowed region, and only 0.726% were in the questionable region. Generally, considering the proportion of residues in the allowed dihedral angle distribution region of a qualified model is no less than 90% (Supplementary Fig. 2), so the RANKL trimer model we constructed in this study can be considered to be reasonable^[8]^. Furthermore, Verify3D suggested that 94.02% of the residues in the RANKL trimer model had an average 3D–1D score ≥0.2, which exceeds the set threshold of Verify3D (80%), indicating that the side-chain distribution of this model was reasonable and its stereo structure was compatible with its primary structure^[9]^ (Supplementary Fig. 3). The above data thus indicated that the modeling structure of the RANKL trimer was of good quality and could be further studied.

*The analysis of potential ZOL binding sites*

In this study, we generated a total of 20 potential binding conformations, as shown Supplementary Figure 5. These ligand-binding conformations were mainly distributed in four sites of the RANKL trimer, most of which bound in the trimer channel, followed by binding in the loop regions of structure connection at the bottom of the trimer channel. By observation of the ligand binding conformations, we found that ZOL was more likely to dock around the channel than on the protein surface, which indicating that the internal hydrophobic channel of the RANKL trimer was more likely to be potential drug binding sites.

**Supplemental References**

[1] Koch FP, Merkel C, Ziebart T, Smeets R, Walter C, Al-Nawas B. Influence of bisphosphonates on the osteoblast RANKL and OPG gene expression in vitro. Clin Oral Investig. 2012. 16(1): 79-86.

[2] Luan X, Lu Q, Jiang Y, et al. Crystal structure of human RANKL complexed with its decoy receptor osteoprotegerin. J Immunol. 2012. 189(1): 245-52.

[3] Zakłos-Szyda M, Budryn G, Grzelczyk J, Pérez-Sánchez H, Żyżelewicz D. Evaluation of Isoflavones as Bone Resorption Inhibitors upon Interactions with Receptor Activator of Nuclear Factor-κB Ligand (RANKL). Molecules. 2020. 25(1).

[4] Radwan A, Mahrous GM. Docking studies and molecular dynamics simulations of the binding characteristics of waldiomycin and its methyl ester analog to Staphylococcus aureus histidine kinase. PLoS One. 2020. 15(6): e0234215.

[5] Hintze BJ, Lewis SM, Richardson JS, Richardson DC. Molprobity's ultimate rotamer-library distributions for model validation. Proteins. 2016. 84(9): 1177-89.

[6] Eisenberg D, Lüthy R, Bowie JU. VERIFY3D: assessment of protein models with three-dimensional profiles. Methods Enzymol. 1997. 277: 396-404.

[7] da Silva RC, Siqueira AS, Lima A, et al. In silico characterization of a cyanobacterial plant-type isoaspartyl aminopeptidase/asparaginase. J Mol Model. 2018. 24(5): 108.

[8] Yu Z, Kang L, Zhao W, et al. Identification of novel umami peptides from myosin via homology modeling and molecular docking. Food Chem. 2021. 344: 128728.

[9] Guo S, Yang J, Lei Y, et al. Which species does the virus like most: Binding modes study between SARS-CoV-2 S protein and ACE2 receptor. J Mol Graph Model. 2021. 105: 107893.

**Supplementary Figures and Tables:**


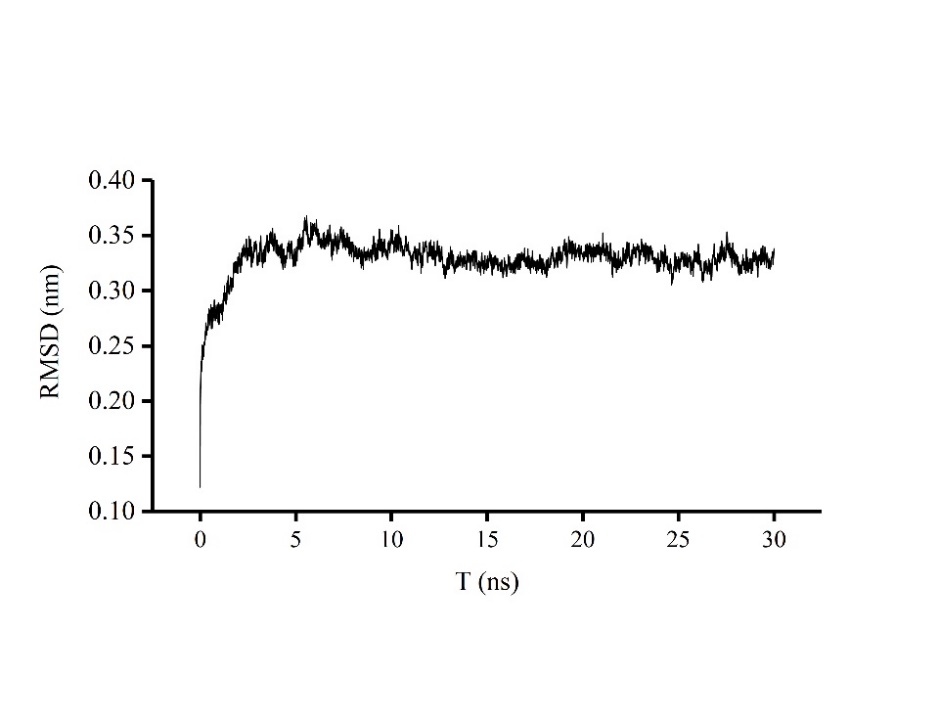


**
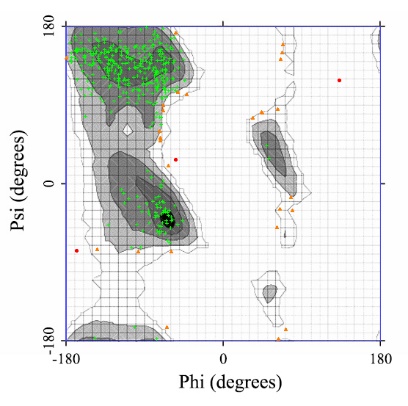
Supplementary Figure 1** RMSD graph of the original RANKL trimer structure of the 30-ns MD simulation.

**Supplementary Figure 2** Ramachandran plot of RANKL trimer model. Highly preferred conformations, shown as green crosses, are located in the regions colored in shades of gray. Allowed conformations, shown as brown triangles, are located in the regions with black grids. Questionable conformations, shown as red circles, are located in the regions with a light-gray grid.


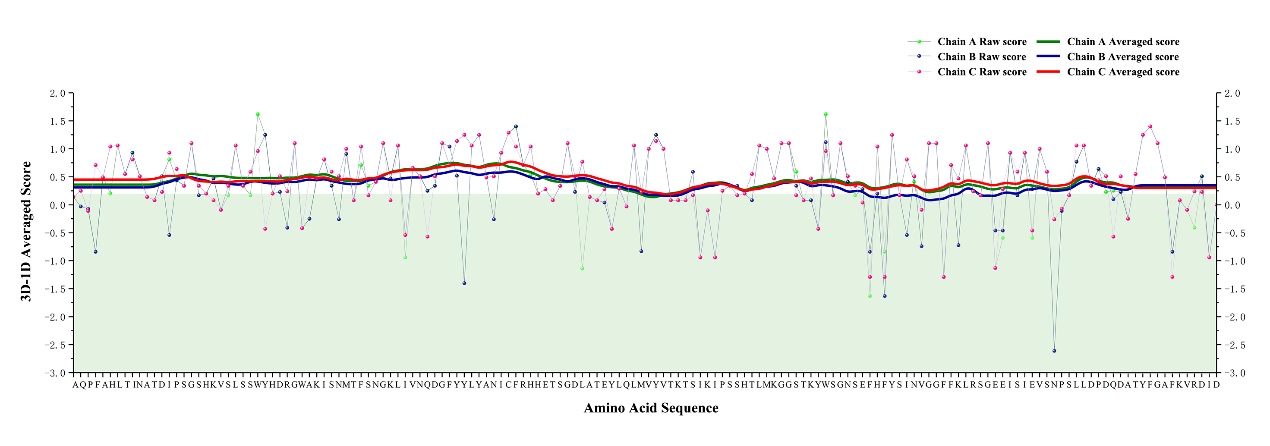
**Supplementary** **Figure 3** Verify3D profile of the computed RANKL trimer model. Green dots represent the residues of chain A, blue dots represent the residues of chain B, and pink dots represent the residues of chain C. The lines of different colors represent the 3D–1D averaged scores of the residues in each chain, and the colors of the lines correspond to the colors of the balls, indicating each chain of the model. The region colored in light green represents 3D–1D averaged scores less than 0.2.


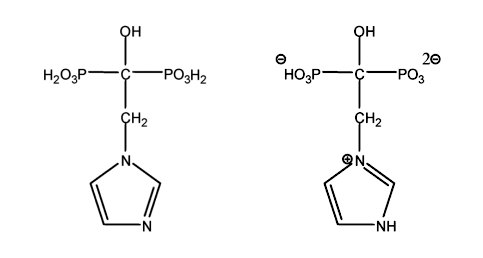


**Supplementary** **Figure 4** The 2D structure of ZOL. The plot on the left represents the molecular structure of ZOL, the plot on the right represents the conformation of ZOL in ionization state at pH = 7.4.


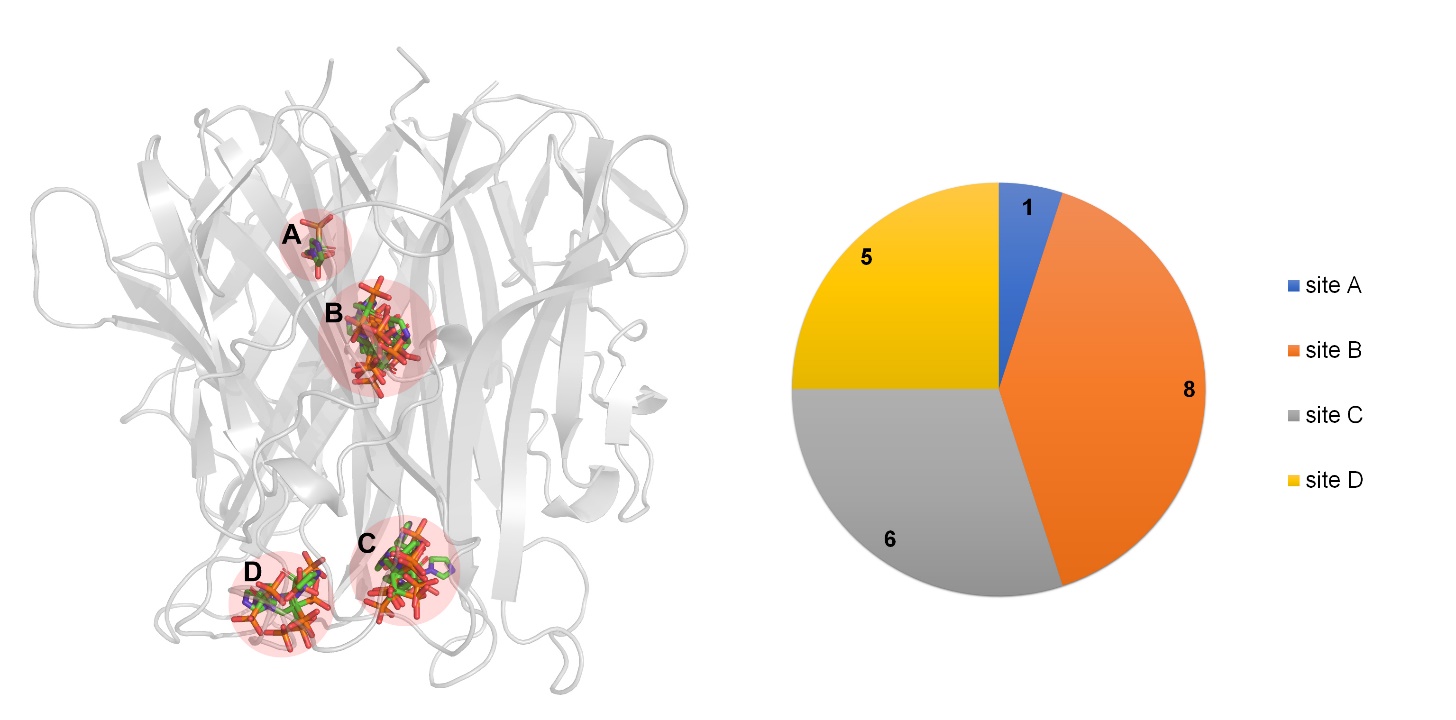


**Supplementary** **Figure 5** The distribution of potential ZOL binding sites

| Receptor (RANKL) | | | Ligand (ZOL) | | Distance(Å) |
| --- | --- | --- | --- | --- | --- |
| Chain A | Tyr215-OH | O6 | | 2.72 | |
|  | Val277-O | O4 | | 3.01 | |
| Chain B | Tyr215-OH | O6 | | 2.90 | |
|  | Asn276-OD1 | O2 | | 2.53 | |
|  | Asn276-OD1 | O3 | | 2.88 | |
|  | Asn276-ND2 | O2 | | 3.01 | |
| Chain C | Tyr215-OH | O6 | | 2.82 | |
|  | Asn276-OD1 | O3 | | 2.82 | |
|  | Asn276-OD1 | O7 | | 2.69 | |

**Supplementary Table 1** Hydrogen bonding interaction between RANKL and ZOL

| Complex | $\boldsymbol{\Delta E}_{\mathbf{vdw}}$ | $\boldsymbol{\Delta E}_{\mathbf{ele}}$ | $\boldsymbol{\Delta G}_{\mathbf{pol}}$ | $\boldsymbol{\Delta G}_{\mathbf{nonpol}}$ | $\boldsymbol{\Delta G}_{\mathbf{binding}}$ |
| --- | --- | --- | --- | --- | --- |
| RANKL–ZOL | −138.137 ± 3.79 | −123.500 ± 3.04 | 204.041 ± 1.08 | −13.068 ± 0.04 | −70.665 ± 2.62 |

**Supplementary Table 2** Results of the average binding free-energy calculations of RANKL and ZOL from the MM-PBSA method (kJ/mol). The energy values were expressed as mean ± SD.

| Receptor | | Binding free energy (kJ/mol) |
| --- | --- | --- |
| Chain A | VAL-277 | -7.71919 ± 0.94799 |
|  | GLY-278 | -6.56383 ± 0.48927 |
|  | TYR-217 | -4.4888 ± 0.56077 |
|  | TYR-215 | -3.09835 ± 0.33621 |
|  | GLY-279 | -1.46401 ± 0.33943 |
| Chain B | GLY-278 | -5.49545 ± -1.68767 |
|  | VAL-277 | -4.64557 ± 1.23917 |
|  | TYR-217 | -3.19083 ± 0.73568 |
|  | TYR-215 | -2.3193 ± 0.46418 |
|  | GLY-279 | -1.68767 ± 0.36574 |
| Chain C | TYR-215 | -4.92687 ± 1.13427 |
|  | VAL-277 | -3.21917 ± 0.80373 |
|  | ASN-276 | -1.86002 ± 0.48768 |
|  | GLY-279 | -1.0978 ± 0.22806 |
|  | ASP-317 | -1.06348 ± 0.2925 |

**Supplementary Table 3** The average binding free energy of the top five residues of each chain. The energy values were expressed as mean ± SD.
